# Serum Uric Acid Levels were Dynamically Coupled with Hemoglobin A1c in the Development of Type 2 Diabetes

**DOI:** 10.1038/srep28549

**Published:** 2016-06-22

**Authors:** Fengjiang Wei, Baocheng Chang, Xilin Yang, Yaogang Wang, Liming Chen, Wei-Dong Li

**Affiliations:** 1 Center of Basic Medical Sciences, Tianjin Medical University, 22 Qixiangtai Road, Tianjin, 300070, P.R. China; 2 Metabolic Diseases Hospital, Tianjin Medical University, Tianjin, 300070, P.R. China; 3 Department of Epidemiology and Biostatistics, School of Public Health, Tianjin Medical University, 22 Qixiangtai Road, Tianjin, 300070, P.R. China; 4 School of Public Health, Tianjin Medical University, 22 Qixiangtai Road, Tianjin, 300070, P.R. China

## Abstract

The aim of the study was to decipher the relationship between serum uric acid (SUA) and glycated hemoglobin A1c (HbA1c) or fasting plasma glucose (FPG) in both type 2 diabetes mellitus (T2DM) patients and normal subjects. A total of 2,250 unrelated T2DM patients and 4,420 Han Chinese subjects from a physical examination population were recruited for this study. In T2DM patients SUA levels were negatively correlated with HbA1c (*r*_*s*_ = *−*0.109, *P* = 0.000) and 2 h plasma glucose levels (*r*_*s*_ = *−*0.178, *P* = 0.000). In the physical examination population, SUA levels were inversely correlated with HbA1c (*r*_*s*_ = *−*0.175, *P* = 0.000) and FPG (*r*_*s*_ = *−*0.131, *P* = 0.009) in T2DM patients but positively correlated with HbA1c (*r*_*s*_ = 0.040, *P* = 0.012) and FPG (*r*_*s*_ = 0.084, *P* = 0.000) in normal-glucose subjects. Multivariate analyses showed that HbA1c was significantly negatively associated with HUA both in T2DM patients (OR = 0.872, 95% CI: 0.790~0.963) and in the physical examination T2DM patients (OR = 0.722, 95% CI: 0.539~0.968). Genetic association studies in T2DM patients showed that alleles of two glucose-uric acid transporter genes, *ABCG2* and *SLC2A9* were significantly associated with SUA levels (*P* < 0.05). SUA level is inversely correlated with HbA1c in T2DM patients but positively correlated with HbA1c in normal-glucose subjects. The reverse transporting of uric acid and glucose in renal tubules might be accounted for these associations.

Serum uric acid (SUA) is the final oxidation product of purine metabolism in circulation. Hyperuricemia (HUA), or elevated SUA levels, has been considered not only an independent risk factor for cardiovascular diseases (CVDs) but also a factor in the development of metabolic diseases^1^. Diseases associated with HUA in humans include hypertension, chronic kidney disease, and type 2 diabetes mellitus (T2DM). Interestingly, low SUA levels have also been found to be associated with diseases of neurological origin, such as Parkinson’s disease, multiple sclerosis, and Alzheimer’s disease^2^. Elevated SUA levels (i.e., HUA) have been associated with MetS and CVD morbidity and mortality. HUA and MetS were both associated with adverse renal outcome.

Hemoglobin A1c (HbA1c) has been recommended by the American Diabetes Association as an optional assay for the diagnosis of T2DM and also for identifying individuals at increased risk of T2DM. HbA1c reflects an average blood glucose level over the past 3 months. Thus, HbA1c levels provide a longer-term picture of blood glucose status as an exposure variable. However, the relationship between SUA and HbA1c/FPG was not completely consistent: many studies showed a positive correlation between SUA and fasting plasma glucose (FPG) while others suggested an inverse relationship^3^,^4^,^5^. Recent studies (including ours) found that uric acid-hexose transporters account for significant amount of genetic variance of SUA^6^,^7^,^8^, thus, we suspected that the SUA-FPG paradox actually is a result of glucose-uric acid coupled transporting. Instead of a cross section study for SUA-HbA1c/FPG in T2DM patients, it is necessary to evaluate the dynamic change of the SUA and FPG levels in different stages of hyperglycemia.

In this study, we examined the correlation between SUA and HbA1c/FPG levels in T2DM patients and subjects with normal FPG. Besides, we also considered the influence of renal function (measured by estimated glomerular filtration rates (eGFR)) on the SUA-HbA1c correlation. Interestingly, we found the SUA level seems coupled with FPG and HbA1c in both T2DM and normal individuals, although the correlations were not in the same direction. SUA levels changed with FPG, however, the SUA-FPG coupling vanished in T2DM patients with impaired renal function. We also found genotype-phenotype associations among two hexose-uric acid transporter gene polymorphisms and SUA. These results suggested a plausible mechanism that the glucose-SUA coupled transportation may account for the dynamic SUA level change during different stages of T2DM.

## Results

### Baseline characteristics

The T2DM patients comprised 1,218 (54.1%) men (median (±*Q*) 57.00 ± 14.00 years of age) and 1,032 (45.9%) women (61.00 ± 15.00 years of age). In the physical examination population, 2550 (71.9%) men (62.87 ± 15.62 years of age) and 999 (28.1%) women (61.10 ± 15.62 years of age) had normal glucose, and 722 (82.9%men (69.62 ± 12.92 years of age) and 149 (17.1%) women (75.79 ± 22.78 years of age) were T2DM. HUA prevalence in the physical examination population was 16.20% (men, 17.67%; women, 12.02%) and was more frequent in men (*χ*^2^ = 19.943, *P* = 0.000). HUA prevalence in the T2DM patients was 17.25% (men, 14.86%; women, 20.06%) and was more frequent in women (*χ*^2^ = 10.576, *P* = 0.001).

In the T2DM patients (Table 1), HUA and normal subjects did not significantly differ in DBP (*P* = 0.298), ALT (*P* = 0.388), GGT (*P* = 0.173), HDL (*P* = 0.110), LDL (*P* = 0.415),FBG (*P* = 0.601), or plasma glucose 2 h after an oral glucose tolerance test (P2hG; *P* = 0.262), Furthermore, between the T2DM patients with normal SUA and HUA, a gradual and significant increasing trend was observed in age (*P* = 0.001), BMI (*P* = 0.000),SBP (*P* = 0.000), AST (*P* = 0.000), BUN (*P* = 0.000), serum CR (*P* = 0.000), SUA (*P* = 0.000), TC (*P* = 0.007), TG (*P* = 0.000), VLDL (*P* = 0.000), FINS (*P* = 0.000), P2INS (*P* = 0.000), and HOMA-IR (*P* = 0.000), but a decreasing trend in eGFR (*P* = 0.000) and glycated HbA1c (*P* = 0.001).

In normal-glucose subjects in the physical examination population (Table 2), a gradual and significant increasing trend was observed from normal to HUA subjects in age (*P* = 0.001), BMI (*P* = 0.000), SBP (*P* = 0.000), DBP (*P* = 0.019), BUN (*P* = 0.000), serum CR (*P* = 0.000), SUA (*P* = 0.000), FBG (*P* = 0.001), glycated HbA1c (*P* = 0.000), and TG (*P* = 0.000) but a decreasing trend in eGFR (*P* = 0.000) and HDL (*P* = 0.000).

In T2DM patients of physical examination population (Table 2), there was no significant difference between HUA and normal-SUA subjects in SBP (*P* = 0.413), DBP (*P* = 0.719), BUN (*P* = 0.313), and HDL (*P* = 0.075). A gradual and significant increasing trend was observed in age (0.025), BMI (*P* = 0.002), serum CR (*P* = 0.002), SUA (*P* = 0.000), and TG (*P* = 0.000) but a decreasing trend in eGFR (*P* = 0.000), FBG (*P* = 0.028), and glycated HbA1c (*P* = 0.031).

### Correlation between characteristics and SUA

A Spearman correlation analysis was used to estimate correlation between clinical characteristics and SUA in T2DM patients and the physical examination population. SUA levels in T2DM patients were positively correlated with BMI, both SBP and DBP, renal function (serum CR, BUN), blood lipids (TG, VLDL), blood glucose, and insulin resistance index (FINS, P2INS, HOMA-IR; *P*-values < 0.05) and negatively correlated with HDL, glycated HbA1c, P2hG, and eGFR (*P*-values < 0.05) (Table 3). We found no significant correlation between SUA and age, TC, LDL, or FPG.

In normal-glucose subjects in the physical examination population, SUA levels were positively correlated with BMI, both SBP and DBP, renal function (serum CR, BUN), blood lipid (TG), FBG, and glycated HbA1c (*P*-values < 0.05) and negatively correlated with eGFR and HDL (*P*-values < 0.05) (Table 4). We found no significant correlation between SUA and age. In T2DM subjects, SUA levels significantly positive correlated with BMI, DBP, renal function (serum CR, BUN), and blood lipid (TG; *P*-values < 0.05) and negatively correlated with eGFR, FBG, glycated HbA1c, and HDL (*P*-values < 0.05) (Table 4). We found no significant correlation between SUA,age, or SBP.

To investigate effects of impaired renal function on uric acid phenotype correlations, we divided our cohort by normal (≥90 ml/min/1.73 m^2^) and abnormal (<90 ml/min/1.73 m^2^) eGFR. Most correlations listed in Tables 3 and 4 were relatively unchanged with normal eGFR.

### Multivariate Analyses

We carried out binary logistic analyses to assess the relationship between HbA1c/FPG and HUA. In the multivariate models, the presence of HUA was a dependent parameter. Independent parameters were associated with the presence of HUA in univariate analysis with a *p* value of <0.05 (Tables 5 and 6). All the independent parameters were pooled together in the analysis, and adjusted *ORs* were calculated with the stepwise method.

At baseline, in an unadjusted model (Table 5, models 1 and 7), HbA1c was negatively correlated with HUA both in T2DM patients and the physical examination population (T2DM). Hazard ratios for HUA were 0.866 (95% CI, 0.825~0.952) and 0.626 (95% CI, 0.434~0.902), respectively; after adjusting for age and gender (Table 5, models 2 and 8), they were 0.897 (95% CI, 0.833~0.965) and 0.650 (95% CI, 0.462~0.914), respectively. The negative association was also significant after further adjustment for multiple metabolic parameters (Table 5, models 3 and 9), with hazard ratios of 0.872 (95% CI, 0.790~0.963) and 0.722 (95% CI, 0.539~0.968), respectively (*P* < 0.05). In normal-glucose subjects in the physical examination population, the presence of HUA was positively associated with HbA1c. In an unadjusted model (Table 5, model 4), the hazard ratio for HUA was 1.352 (95% CI, 1.101~1.662), but in the adjustment models (Table 5, models 5 and 6), the positive association no longer existed.

In the physical examination population (T2DM), FPG was negatively correlated with HUA (Table 6, models 7). Hazard ratios for HUA were 0.986 (95% CI, 0.973~0.999). After adjusting for age, gender (Table 6, models 8) and multiple metabolic parameters (Table 6, models 9), the negative association was also significant, with hazard ratios of 0.971 (95% CI, 0.949~0.993) and 0.610 (95% CI, 0.419~0.887), respectively (*P* < 0.05).

### Genetic association studies

We carried out association studies among single nucleotide polymorphisms (SNPs) of glucose-uric acid transporter genes and SUA levels in 2250 T2DM patients. Alleles of two glucose-uric acid transporter genes, *ABCG2* (rs2231142) and *SLC2A9* (rs7660895 and rs1014290), were significantly associated with SUA (*P* < 0.05; Table 7). The CC genotype of rs2231142, the AA genotype of rs7660895, and the CC genotype of rs1014290 were associated with lower SUA levels than their counterpart alleles.

## Discussion

Currently, gout and renal disorders were considered main consequences of HUA, and SUA is recognized as a potential risk factor for hypertension, stroke, and CVDs^9^. A large number of studies have linked HUA with hypertension, CVD, and T2DM. An SUA increase of 59.5 *μ*mol/ results in a 60% increase in risk for developing T2DM^10^. The present study demonstrated that HUA prevalence in a physical examination population was 16.20% (men, 17.67%; women, 12.02%) and in T2DM patients was 17.24% (men, 14.86%; women, 20.06%). Qiu *et al*. reported that an HUA prevalence level of 13.7% (21.1% in men and 7.9% in women) in two northern provinces of China^11^. The adjusted prevalence of HUA among Chinese adults in 2009~2010 was 8.4% (men, 9.9%; women, 7.0%)^12^. Compared with these results, our study population had a higher prevalence of HUA.

The prevalence of diabetes has been increasing worldwide, and the total number of people with diabetes has been projected to rise from 94 million in 2003 to 333 million in 2025^13^. Elevated SUA levels predict the onset of T2DM. Approximately 1 in 11 cases of new T2DM and approximately 8.7% of all new cases of T2DM were statistically attributed to HUA^14^,^15^. Ogbera *et al*. reported a 25% prevalence of HUA in Nigerian patients with T2DM. SUA increases with age and may occur more in women, especially after attainment of menopause^16^. Among Chinese T2DM patients with central obesity, HUA prevalence was 36.1% in women and 28.4% in men^17^.

Glycated HbA1c is widely used as a clinical parameter of chronic glycemic control. HbA1c is an easily determined measure of mean glucose level over the previous 2~3 months and is the recommended method for assessing the effectiveness of therapies^15^. HbA1c has been proposed as a diagnostic tool to identify people with undiagnosed T2DM or who are at risk of T2DM. HbA1c does not require fasting, has better pre-analytical stability, and reflects long-term glycemic exposure better than do current diagnostic tests based on fasting or post load glucose measures^18^. One of the interesting findings of our present study is the inverse association of SUA with diabetic parameters (HbA1c, FPG). The T2DM subjects showed a negative association between SUA and HbA1c (*P* = 0.000). The T2DM subjects in the physical examination population showed a negative association between SUA with FBG (*P* = 0.009) and HbA1c (*P* = 0.000). In contrast, normal glucose subjects in the physical examination population showed a positive association between SUA with FBG (*P* = 0.000) and glycated HbA1c (*P* = 0.012). Multivariate analyses showed that HUA was significantly associated with low HbA1c both in T2DM subjects (OR = 0.872, 95% CI: 0.790~0.963) and in the physical examination population (T2DM) (OR = 0.722, 95% CI: 0.539~0.968).

Nan H. *et al*. reported that SUA levels tended to increase with increasing FPG levels in non-diabetic individuals but decreased in diabetic individuals^3^. In a nationally representative sample of US men and women, SUA levels and the frequency of HUA increased with moderately increasing levels of HbA1c (6~6.9%) and then decreased with further increasing levels of HbA1c (a bell-shaped relation); SUA levels showed a similar bell-curved relation with FBG, and there was a positive association between SUA and HbA1c levels <7% but an inverse association between SUA and T2DM (or HbA1c ≥ 7%)^19^. Wang *et al*. found that the risk of HUA decreased 0.928-fold for every 1% increase in HbA1c and 0.962-fold for every 1 mmol/l increase in FPG^17^. Li *et al*. reported that SUA was negatively correlated with HbA1c (correlation coefficient = *−*0.24, *p* < 0.001) and FPG levels (correlation coefficient = *−*0.26, *p* < 0.001) in patients with T2DM^4^. But Fan *et al*. found that SUA in individuals with IFG and/or IA1c was positive associated with 2-h PG, independent of FPG and HbA1c, the result was somewhat contrary to our findings^9^. The inverse relationship between SUA and diabetic parameters (HbA1c, FPG) observed in these diabetic subjects may be caused by increased renal excretion of uric acid in the presence of hyperglycemia. It is well known that with increasing duration of diabetes, which is accompanied by worsening of beta cell function and deterioration of glycemic control, the rate of renal filtration increases gradually. The hyperfiltration state caused by hyperglycemia promotes the excretion of uric acid, which partly explains the inverse relationship between SUA and diabetic parameters^4^.

Mechanisms that relate SUA to T2DM are still unclear, but several have been suggested. Animal experiments have shown that HUA may induce endothelial dysfunction by inhibiting nitric oxide (NO) bioavailability. Because insulin depends on NO for stimulation of glucose uptake, this may result in inhibited glucose uptake. Therefore, HUA may play an important role in the development or worsening of insulin resistance. Furthermore, a direct effect on adipocytes has been suggested: in mouse adipocytes, uric acid induced a proinflammatory state^20^.

The positive SUA-FPG (and HbA1c) correlation in normal individuals and the inverse correlation in T2DM patients suggested a regulatory SUA mechanism that coupled with FPG levels. Indeed, gene polymorphisms of two hexose-uric acid transporters in renal tubules (*SLC2A9* and *ABCG2*) are associated with HUA in genome-wide association studies^21^,^22^. We further genotyped *SLC2A9* and *ABCG2* gene polymorphisms in our populations, results showed that individuals with different *SLC2A9* and *ABCG2* genotypes indeed had different SUA levels. Our study showed the associations between SUA level and SNPs in *ABCG2* and *SLC2A9* in Chinese Han T2DM patients. Alleles rs7660895 and rs1014290 of the *SLC2A9* gene and rs2231142 of the *ABCG2* gene were significantly associated with SUA levels (*P* < 0.05). The CC genotype of rs2231142, the AA genotype of rs7660895, and the CC genotype of rs1014290 were associated with lower uric acid levels than their counterpart alleles. Recently, Giri *et al*. found that *SLC2A9* and *ABCG2* gene variants were associated with SUA level in Indians, differences in effect sizes of *SLC2A9* and *ABCG2* polymorphisms were observed between healthy subjects and type 2 diabetes patients^23^. Liu *et al*. showed that the *SLC2A9* gene SNP rs1014290 was associated with the decreased risk of type 2 diabetes, serum glucose level, and SUA in Han Chinese^24^. *SLC2A9* and *ABCG2* are most strongly associated with regulating SUA concentration. *ABCG2* contributes not only to renal urate excretion but also to gut urate excretion via intestinal and biliary secretion in humans^25^. *SLC2A9* was a transporter for both fructose and urate. Yang *et al*. reported that the loci of *ABCG2* and *SLC2A9* could explained 1.09% and 1.03% of the variation of SUA levels respectively^26^.

For those individual with impaired renal function (eGFR decreased, Table 3), the correlation between SUA and FPG vanished, it also suggested the glucose-uric acid coupling might dependent on hexose-uric acid transporters at proximal renal tubules. Like the regulation of the blood glucose, the SUA level might be precisely adjusted via the renal uric acid-hexose transporters. The increased SUA might be an early signal of insulin resistance, the inverse correlation between SUA and HbA1c (FPG) continues until the renal function was severely damaged by diabetic nephropathy.

## Materials and Methods

### Participants

We recruited 2,250 unrelated Han Chinese T2DM patients in Tianjin, China (2011~2014). Diabetes mellitus was defined upon World Health Organization criteria (fasting plasma glucose ≥ 7.0 mmol/L, and/or 2-h oral glucose tolerance tests (OGTT) ≥ 11.1 mmol/L), or the use of anti-diabetic medicine. All the subjects were unrelated Han Chinese receiving treatment at the Metabolic Disease Hospital of Tianjin Medical University, the General Hospital of Tianjin Medical University, the Tianjin People’s Hospital, and the Eye Hospital of Tianjin Medical University. We have also collected 4,420 Han Chinese subjects from a physical examination population in 2014: 871 were T2DM patients (fasting plasma glucose ≥ 7.0 mmol/L) and 3549 had normal fasting plasma glucose (FPG) levels (fasting plasma glucose < 6.1 mmol/L). Subject recruiting protocols were reviewed and approved by the Human Ethics Committee of Tianjin Medical University. Subjects gave written informed consent prior to participating in this study. All experiments were performed in accordance with relevant guidelines and regulations.

### Measurement

All subjects were measured twice for height and weight (using identical standardized anthropometric scales), and body mass index (BMI) was calculated (kg/m^2^). Diabetes mellitus was defined upon World Health Organization criteria (fasting plasma glucose ≥ 7.0 mmol/L, and/or 2-h oral glucose tolerance tests (OGTT) ≥ 11.1 mmol/L), or the use of anti-diabetic medicine. Fasting blood samples were drawn after 12 h of fasting followed by an OGTT (75 g glucose were given) to evaluate glucose tolerance status and OGTT-related insulin release (samples for measurement of plasma glucose and serum insulin were drawn at 0, 30, 90 and 120 min). Fasting plasma glucose (FPG), plasma glucose 120 min post- OGTT (P2hG), fasting serum insulin (FINS), serum insulin 120 min post-OGTT (P2INS) were collected. SUA levels were measured by enzymatic methods (Chemistry Analyzer Au2700, Olympus Medical Engineering Company, Japan). Blood pressure (BP) was measured by a physician using a mercury sphygmomanometer, and hypertension was defined as systolic BP (SBP) at least 140 mmHg and/or diastolic BP (DBP) at least 90 mmHg, and/or use of antihypertension medication. Total cholesterol (TC), high-density- lipoprotein cholesterol (HDL), low-density-lipoprotein cholesterol (LDL), triglycerides (TG), serum creatinine (SCR), blood urea nitrogen (BUN), galactosyl glucosyltransferase (GGT), alanine aminotransferase (ALT), Aspartate transaminase (AST) and glycated hemoglobin (HbA1c) were measured using the Sysmex Chemix-180 automatic biochemical analysis device (Sysmex Infosystems, Kobe, Japan). Insulin was measured by an enzymatic luminescence technique. HUA was defined as an SUA concentration ≥ 420 *μ*mol/l in men and ≥360 *μ*mol/l in women^27^. Values of estimated glomerular filtration rate (eGFR; ml/min/1.73 m^2^) were calculated by using the equation proposed by investigators in the Chronic Kidney Disease Epidemiology Collaboration^28^. Insulin resistance was estimated using the homeostasis model assessment index-insulin resistance (HOMA-IR): HOMA-IR = [fasting insulin (*μ*IU/ml) × fasting glucose (mmol/l)]/22.5^29^.

### Genotyping for glucose-uric Acid transporter genes

We carried out association studies among single nucleotide polymorphism (SNPs) of two renal tubular glucose-uric acid transporter genes, *SLC2A9* and *ABCG2*, and serum uric acid levels. Genomic DNA samples were extracted from peripheral whole blood samples using the high-salt method. We selected 3 candidate SNPs from *SLC2A9* (rs13129697, rs7660895, rs1014290) and 1 candidate SNPs from *ABCG2* (rs2231142), genotyping was performed by primer extension of multiplex products with detection by matrix-assisted laser desorption time-of-flight mass spectrometry.

### Statistical analysis

The basic characteristics of the sample were described by descriptive statistics. The clinical characteristics between normal and HUA groups were analyzed using the non-parametric test in the continuous variables. The chi-square test was used to compare the differences in percentages in the dichotomous variables. Spearman’s correlation coefficients were calculated to describe the association between variables. A non-conditional logistic regression model was used to examine which variables were statistically significant risk factors in certain groups in relation to and adjusted for the relevant confounders using bivariate analysis. Odds ratio (OR) and 95% confidence interval (CI) for HUA were calculated. For statistical inference, a bilateral *P*-value of <0.05 was considered statistically significant. All statistical analyses were carried out using SPSS statistical software, version 17.0 (SPSS Inc., Chicago, IL, USA) for Windows. All phenotypes were documented in a Filemaker Pro database. An association analysis was performed using PLINK (http://pngu.mgh.harvard.edu/~purcell/plink/). We also compared the SUA levels between different genotypes by ANOVA (SPSS Inc., Chicago, IL, USA).

## Conclusions

We have found that SUA level is inversely associated with HbA1c in T2DM patients but positively correlated with HbA1c in normal-glucose subjects. Genetic association studies in T2DM patients showed that two glucose-uric acid transporter genes, *ABCG2* and *SLC2A9*, were significantly associated with SUA in T2DM patients. In those individuals with impaired renal function, the SUA-FPG coupling vanished. Seems the reverse transporting of uric acid and glucose in renal tubules might be accounted for the associations between SUA and HbA1c, the SUA-T2DM association needs a reappraisal in the future epidemiology studies. Additional attention should be given to healthy lifestyles and early interventions to control obesity and lipid abnormalities, to effectively reduce HUA risk and avoid the occurrence of gout. In future research, we plan to investigate clinical outcomes of individuals with elevated SUA levels in our cohort and to test genetic backgrounds related to HUA in general populations.

## Additional Information

**How to cite this article**: Wei, F. *et al*. Serum Uric Acid Levels were Dynamically Coupled with Hemoglobin A1c in the Development of Type 2 Diabetes. *Sci. Rep.* **6**, 28549; doi: 10.1038/srep28549 (2016).

## Figures and Tables

**Table 1:** Basic characteristics of the subjects with type 2 diabetes mellitus (*M* ± *Q*).

**Characteristic**	**Total**	**Normal**	**HUA**	***Z*****value**	***P*****value**
*N* (male/female)	2250 (1218/1032)	1862 (1037/825)	388 (181/207)	10.576	0.001
Age (years)	59.00 ± 15.00	58.00 ± 14.00	61.00 ± 19.00	−3.274	0.001
BMI (kg/m^2^)	25.93 ± 4.85	25.71 ± 4.66	27.10 ± 5.10	−5.881	0.000
SBP (mmHg)	130.00 ± 15.00	130.00 ± 15.00	130.00 ± 20.00	−4.347	0.000
DBP (mmHg)	80.00 ± 10.00	80.00 ± 10.00	80.00 ± 10.00	−1.040	0.298
ALT (IU/l)	20.00 ± 15.15	20.00 ± 14.73	18.95 ± 16.60	−0.862	0.388
AST (IU/l)	18.40 ± 9.00	18.00 ± 8.72	19.60 ± 9.50	−3.726	0.000
GGT (IU/l)	24.60 ± 0.70	24.00 ± 20.40	25.50 ± 21.45	−1.362	0.173
eGFR (ml/min per 1.73 m^2^)	97.38 ± 45.21	100.27 ± 43.41	74.76 ± 48.63	−11.774	0.000
BUN (mmol/l)	5.70 ± 2.39	5.60 ± 2.20	6.91 ± 3.36	−10.592	0.000
SCR (μmol/l)	69.60 ± 29.65	67.50 ± 26.40	86.00 ± 43.70	−11.255	0.000
SUA (μmol/l)	302.20 ± 119.70	283.80 ± 95.30	441.15 ± 78.88	−28.760	0.000
TG (mmol/l)	1.63 ± 1.22	1.56 ± 1.14	1.95 ± 1.45	−6.250	0.000
TC (mmol/l)	5.14 ± 1.56	5.13 ± 1.49	5.28 ± 1.88	−2.707	0.007
HDL (mmol/l)	1.30 ± 0.50	1.30 ± 0.40	1.30 ± 0.40	−1.596	0.110
LDL (mmol/l)	3.13 ± 1.31	3.13 ± 1.26	3.16 ± 1.42	−0.815	0.415
VLDL (mmol/l)	0.60 ± 0.45	0.59 ± 0.40	0.66 ± 0.54	−4.233	0.000
HbA1c (%)	7.70 ± 2.40	7.70 ± 2.40	7.40 ± 1.90	−3.225	0.001
HbA1c (mmol/mol)	64.23 ± 19.72	64.57 ± 19.79	60.63 ± 17.30	3.341	0.001
FPG (mmol/l)	7.70 ± 2.98	7.70 ± 3.00	7.70 ± 2.78	−0.522	0.601
P2hG (mmol/l)	18.10 ± 5.95	18.10 ± 5.85	17.50 ± 6.60	−1.121	0.262
FINS (mIU/l)	8.97 ± 9.90	8.63 ± 9.58	12.70 ± 10.45	−4.419	0.000
P2INS (mIU/l)	35.80 ± 41.95	35.00 ± 39.55	46.10 ± 66.41	−3.547	0.000
HOMA-IR	2.96 ± 3.63	2.72 ± 3.61	4.21 ± 3.73	−3.624	0.000
duration of diabetes (years)	9.00 ± 11.00	9.00 ± 11.00	10.00 ± 11.00	−3.667	0.000

BMI, body mass index; SBP, systolic blood pressure; DBP, diastolic blood pressure; ALT, alanine aminotransferase; AST, aspartate transaminase; GGT, glutamyl transpeptidase; eGFR, estimated glomerular filtration rate; BUN, blood urea nitrogen; SCR, serum creatinine; SUA, serum uric acid; TG, triglycerides; TC, total cholesterol; HDL, high-density lipoprotein-cholesterol; LDL, low-density lipoprotein cholesterol; VLDL,very low-density lipoprotein; HbA1c, glyated hemoglobin; FPG, fasting plasma glucose; P2hG, plasma glucose 120 min after an oral glucose tolerance test; FINS, fasting serum insulin; P2INS, serum insulin 120 min after an oral glucose tolerance test; HOMA-IR, homeostasis model assessment of insulin resistance.

**Table 2:** Basic characteristics of the physical examination population.

**Characteristic**	**Normal glucose**					**T2DM**				
	**Total**	**Normal SUA**	**HUA**	***Z***	***P***	**Total**	**Normal SUA**	**HUA**	***Z***	***P***
*N* (male/female)	3549 (2550/999)	2993 (2098/895)	556 (452/104)	20.072	0.000	871 (722/149)	711 (596/115)	160 (126/34)	2.373	0.123
Age (years)	63.10 ± 25.62	63.10 ± 25.02	63.70 ± 28.02	−2.328	0.000	72.69 ± 24.42	73.71 ± 24.42	68.10 ± 23.00	−2.025	0.025
eGFR (ml/min per 1.73 m^2)^	85.77 ± 21.43	87.21 ± 20.72	76.48 ± 24.13	−14.683	0.000	84.63 ± 20.46	85.60 ± 19.50	78.35 ± 22.85	−3.501	0.000
BMI (kg/m^2^)	24.59 ± 4.24	24.39 ± 4.14	25.91 ± 4.37	−9.684	0.000	26.06 ± 3.84	25.78 ± 4.01	27.08 ± 3.97	−3.090	0.002
SBP (mmHg)	137.00 ± 26.00	136.00 ± 27.00	139.00 ± 25.00	−3.526	0.000	146.00 ± 29.00	146.00 ± 29.00	146.00 ± 25.50	−0.819	0.413
DBP (mmHg)	75.00 ± 18.00	75.00 ± 17.00	77.00 ± 19.00	−2.340	0.019	77.00 ± 18.00	76.50 ± 18.00	77.50 ± 17.50	−0.360	0.719
BUN (mmol/l)	5.10 ± 1.80	5.00 ± 1.60	5.50 ± 2.20	−9.975	0.000	5.60 ± 1.90	5.60 ± 2.00	5.70 ± 1.95	−1.009	0.313
SCR (μmol/l)	80.00 ± 20.00	78.00 ± 19.00	89.00 ± 24.00	−16.753	0.000	82.00 ± 18.00	81.00 ± 16.00	87.00 ± 20.00	−3.063	0.002
SUA (μmol/l)	329.00 ± 109.00	312.00 ± 90.00	451.00 ± 57.00	−38.985	0.000	325.00 ± 105.00	308.00 ± 82.00	451.50 ± 51.50	−12.716	0.000
FPG (mmol/l)	5.20 ± 0.70	5.20 ± 0.70	5.30 ± 0.80	−3.193	0.001	7.90 ± 1.50	8.00 ± 1.50	7.50 ± 1.05	−2.201	0.028
HbA1c (%)	5.70 ± 0.50	5.70 ± 0.50	5.80 ± 0.60	−4.006	0.000	7.30 ± 1.50	7.30 ± 1.50	7.05 ± 1.38	−2.108	0.031
HbA1c (mmol/mol)	39.55 ± 4.51	39.42 ± 4.45	40.21 ± 4.77	−4.057	0.000	56.58 ± 8.74	57.01 ± 9.33	55.12 ± 8.87	−2.336	0.020
TG (mmol/l)	1.22 ± 0.80	1.17 ± 0.74	1.57 ± 1.01	−12.812	0.000	1.49 ± 1.05	1.38 ± 0.96	2.01 ± 1.60	−5.048	0.000
HDL (mmol/l)	1.29 ± 0.51	1.32 ± 0.52	1.18 ± 0.44	−9.478	0.000	1.13 ± 0.38	1.15 ± 0.40	1.08 ± 0.32	−1.777	0.075
**a** Basic characteristics of the physical examination population (men).										
*N* (male)	2550	2098	452	–	–	722	596	126	–	–
Age (years)	62.70 ± 25.62	63.10 ± 25.18	61.10 ± 27.02	−0.074	0.941	69.12 ± 22.67	69.96 ± 22.62	61.48 ± 25.62	−2.129	0.033
eGFR (ml/min per 1.73 m^2)^	85.89 ± 21.01	87.10 ± 20.29	78.06 ± 23.33	−10.88	0.000	85.78 ± 21.29	87.14 ± 20.70	78.88 ± 22.36	−5.076	0.000
BMI (kg/m^2^)	24.99 ± 4.05	24.76 ± 3.85	26.10 ± 4.12	−7.642	0.000	25.90 ± 3.53	25.79 ± 3.52	26.64 ± 3.70	−2.836	0.005
SBP (mmHg)	137.00 ± 25.00	137.00 ± 25.00	138.00 ± 23.00	−0.877	0.380	146.00 ± 27.00	146.00 ± 29.00	144.00 ± 23.00	−0.514	0.607
DBP (mmHg)	77.00 ± 17.00	77.00 ± 16.00	77.00 ± 18.00	−1.237	0.216	78.00 ± 18.00	78.00 ± 19.00	79.00 ± 16.00	−0.720	0.472
BUN (mmol/l)	5.20 ± 1.80	5.20 ± 1.70	5.60 ± 2.10	−6.043	0.000	5.40 ± 1.80	5.30 ± 1.80	5.60 ± 1.90	−2.080	0.038
SCR (μmol/l)	84.00 ± 16.00	83.00 ± 15.00	91.00 ± 23.00	−12.09	0.000	83.00 ± 17.00	81.50 ± 16.00	89.50 ± 21.00	−5.971	0.000
SUA (μmol/l)	347.00 ± 101.00	332.00 ± 81.00	457.00 ± 53.00	−33.43	0.000	339.00 ± 107.00	321.00 ± 84.50	467.50 ± 51.75	−17.66	0.000
FPG (mmol/l)	5.20 ± 0.60	5.20 ± 0.60	5.30 ± 0.60	−2.252	0.026	6.80 ± 1.50	6.85 ± 1.53	6.80 ± 1.10	−2.101	0.031
HbA1c (%)	5.70 ± 0.60	5.70 ± 0.50	5.75 ± 0.50	−2.128	0.033	6.50 ± 1.20	6.60 ± 1.20	6.50 ± 1.03	−2.018	0.041
TG (mmol/l)	1.23 ± 0.82	1.18 ± 0.75	1.51 ± 1.00	−9.456	0.000	1.38 ± 1.01	1.32 ± 0.91	1.98 ± 1.54	−7.404	0.000
HDL (mmol/l)	1.24 ± 0.43	1.26 ± 0.45	1.16 ± 0.40	−5.945	0.000	1.13 ± 0.39	1.15 ± 0.38	1.04 ± 0.34	−4.025	0.000
**b** Basic characteristics of the physical examination population (women).										
*N* (female)	999	895	104	–	–	149	115	34	–	–
Age (years)	61.10 ± 25.62	60.10 ± 23.60	75.16 ± 25.02	−6.091	0.000	75.75 ± 22.74	76.04 ± 24.00	74.11 ± 22.02	−0.521	0.602
eGFR (ml/min per 1.73 m^2^)	85.59 ± 22.50	87.04 ± 22.05	69.60 ± 23.04	−9.402	0.000	81.82 ± 24.21	84.65 ± 21.18	71.76 ± 28.92	−3.055	0.002
BMI (kg/m^2^)	23.18 ± 3.82	23.05 ± 3.76	24.46 ± 4.97	−3.254	0.001	25.33 ± 5.13	24.30 ± 4.79	27.47 ± 5.03	−3.054	0.002
SBP (mmHg)	132.00 ± 31.00	130.00 ± 30.00	141.00 ± 30.00	−4.855	0.000	146.00 ± 31.00	146.00 ± 34.00	144.50 ± 29.00	−0.787	0.431
DBP (mmHg)	70.00 ± 15.00	70.00 ± 15.00	70.00 ± 18.50	−0.745	0.456	71.00 ± 17.00	72.00 ± 16.00	69.00 ± 17.00	−0.880	0.379
BUN (mmol/l)	4.70 ± 1.60	4.70 ± 1.60	5.50 ± 2.75	−7.156	0.000	5.40 ± 1.83	5.35 ± 2.00	5.55 ± 1.98	−2.223	0.026
SCR (μmol/l)	65.00 ± 13.00	64.00 ± 13.00	76.00 ± 21.00	−9.380	0.000	66.00 ± 15.25	63.00 ± 12.00	73.50 ± 24.75	−3.416	0.001
SUA (μmol/l)	271.00 ± 88.00	262.00 ± 75.00	392.00 ± 53.50	−16.79	0.000	298.50 ± 95.25	281.00 ± 63.25	403.00 ± 70.75	−8.852	0.000
FPG (mmol/l)	5.10 ± 0.60	5.10 ± 0.60	5.20 ± 0.60	−2.102	0.039	6.80 ± 1.10	6.80 ± 1.00	6.70 ± 1.25	−0.817	0.414
HbA1c (%)	5.70 ± 0.50	5.70 ± 0.40	5.80 ± 0.60	−3.905	0.000	6.75 ± 1.10	6.75 ± 1.20	6.60 ± 1.10	−2.032	0.040
TG (mmol/l)	1.15 ± 0.71	1.12 ± 0.66	1.48 ± 0.79	−5.955	0.000	1.44 ± 0.86	1.35 ± 0.79	1.69 ± 1.16	−2.090	0.037
HDL (mmol/l)	1.56 ± 0.55	1.57 ± 0.54	1.40 ± 0.56	−4.061	0.000	1.33 ± 0.43	1.36 ± 0.46	1.26 ± 0.39	−1.178	0.074

Abbreviations are as in Table 1.

**Table 3:** Correlation analyses among serum uric acid levels and clinical characteristics in subjects with type 2 diabetes mellitus.

**Variable**	**All subjects**		**eGFR normal**		**eGFR abnormal**	
	***r***_***s***_	***P***	***r***_***s***_	***P***	***r***_***s***_	***P***
Age (years)	−0.014	0.538	−0.119	0.000	−0.092	0.008
BMI (kg/m^2^)	0.257	0.000	0.297	0.000	0.228	0.000
SBP (mmHg)	0.159	0.000	0.105	0.001	0.084	0.021
DBP (mmHg)	0.092	0.000	0.068	0.030	0.109	0.003
ALT (IU/l)	0.089	0.000	0.178	0.000	0.091	0.029
AST (IU/l)	0.179	0.000	0.199	0.000	0.172	0.000
GGT (IU/l)	0.190	0.000	0.281	0.000	0.131	0.002
BUN (mmol/l)	0.296	0.000	0.132	0.000	0.357	0.000
SCR (μmol/l)	0.410	0.000	0.361	0.000	0.373	0.000
eGFR (ml/min per 1.73 m^2^)	−0.270	0.000	−0.122	0.000	−0.247	0.000
TG (mmol/l)	0.266	0.000	0.309	0.000	0.213	0.000
TC (mmol/l)	0.014	0.540	0.011	0.709	−0.033	0.344
HDL (mmol/l)	−0.155	0.000	−0.184	0.000	−0.155	0.000
LDL (mmol/l)	0.007	0.784	−0.006	0.847	−0.051	0.242
VLDL (mmol/l)	0.217	0.000	0.271	0.000	0.118	0.005
HbA1c (%)	−0.109	0.000	−0.115	0.000	−0.036	0.320
FPG (mmol/l)	−0.035	0.135	−0.018	0.576	−0.022	0.556
P2hG (mmol/l)	−0.178	0.000	−0.221	0.000	−0.140	0.092
FINS (mIU/l)	0.225	0.000	0.218	0.000	0.219	0.010
P2INS (mIU/l)	0.206	0.000	0.207	0.000	0.170	0.052
HOMA-IR	0.190	0.000	0.177	0.000	0.187	0.031
duration of diabetes (years)	0.058	0.009	0.047	0.010	0.026	0.447

Abbreviations are as in Table 1.

**Table 4:** Correlation analyses among serum uric acid and clinical characteristics in the physical examination population.

**Variable**	**Normal glucose**						**T2DM**					
	**All subjects**		**eGFR normal**		**eGFR abnormal**		**All subjects**		**eGFR normal**		**eGFR abnormal**	
	***r***_***s***_	***P***	***r***_***s***_	***P***	***r***_***s***_	***P***	***r***_***s***_	***P***	***r***_***s***_	***P***	***r***_***s***_	***P***
Age (years)	−0.015	0.335	−0.134	0.000	−0.040	0.048	−0.064	0.207	−0.302	0.000	−0.062	0.319
eGFR (ml/min per 1.73 m^2^)	−0.255	0.000	−0.115	0.000	−0.218	0.000	−0.276	0.000	−0.103	0.225	−0.215	0.001
BMI (kg/m^2^)	0.329	0.000	0.369	0.000	0.308	0.000	0.206	0.000	0.254	0.009	0.171	0.013
SBP (mmHg)	0.107	0.000	0.098	0.000	0.071	0.001	−0.015	0.783	−0.008	0.934	−0.065	0.338
DBP (mmHg)	0.166	0.000	0.212	0.000	0.158	0.000	0.106	0.004	0.273	0.003	0.013	0.846
BUN (mmol/l)	0.205	0.000	0.131	0.000	0.187	0.000	0.099	0.048	0.078	0.358	0.016	0.799
SCR (μmol/l)	0.483	0.000	0.467	0.000	0.484	0.000	0.330	0.000	0.307	0.000	0.258	0.000
HbA1c (%)	0.040	0.012	0.003	0.914	0.029	0.146	−0.175	0.000	−0.193	0.022	−0.186	0.003
FPG (mmol/l)	0.084	0.000	0.098	0.000	0.069	0.001	−0.131	0.009	−0.034	0.692	−0.200	0.001
TG (mmol/l)	0.325	0.000	0.391	0.000	0.284	0.000	0.289	0.000	0.373	0.000	0.249	0.000
HDL (mmol/l)	−0.347	0.000	−0.387	0.000	−0.346	0.000	−0.179	0.000	−0.266	0.002	−0.153	0.014

Abbreviations are as in Table 1.

**Table 5:** Odds ratio of HbA1c for incidence of hyperuricemia, and stepwise logistic regression for hyperuricemia.

**Population [*****N*** **(%)]**	**Odds ratio**	**95% CI**	***P*****-value**
T2DM subjects [388 (17.24%)]			
Model1^a^	0.886	0.825~0.952	0.001
Model2^b^	0.897	0.833~0.965	0.003
Model3^c^	0.988	0.981~0.995	0.001
Physical examination population			
Normal glucose [556 (15.67%)]			
Model4^a^	1.352	1.101~1.662	0.004
Model5^b^	1.229	0.974~1.551	0.082
Model6^d^	1.030	0.786~1.350	0.828
T2DM subjects [160 (18.37%)]			
Model7^a^	0.626	0.434~0.902	0.012
Model8^b^	0.650	0.462~0.914	0.013
Model9^e^	0.722	0.539~0.968	0.029

^*^P < 0.05 was considered statistically significant.

^a^Unadjusted baseline values of HbA1c.

^b^Adjusted for age and sex.

^c^Further adjusted for duration of diabetes, BMI, SBP, AST, eGFR, BUN, SCR, TG, TC, VLDL, FINS, P2INS, HOMA-IR. (see Table 1 for abbreviations).

^d^Further adjusted for eGFR, BMI, SBP, DBP, BUN, SCR, FBG, TG, HDL.

^e^Further adjusted for eGFR, BMI, BUN, SCR, FBG, TG.

**Table 6:** Odds ratio of FPG for incidence of hyperuricemia, and stepwise logistic regression for hyperuricemia.

**Population [*****N*** **(%)]**	**Odds ratio**	**95% CI**	***P*****-value**
T2DM subjects [388 (17.24%)]			
Model1^a^	0.983	0.937~1.030	0.468
Model2^b^	0.987	0.941~1.035	0.584
Model3^c^	1.062	0.931~1.212	0.373
Physical examination population			
Normal glucose [556 (15.67%)]			
Model4^a^	1.328	1.067~1.654	0.011
Model5^b^	1.008	1.001~1.014	0.016
Model6^d^	1.020	0.499~2.085	0.956
T2DM subjects [160 (18.37%)]			
Model7^a^	0.986	0.973~0.999	0.030
Model8^b^	0.971	0.949~0.993	0.011
Model9^e^	0.610	0.419~0.887	0.010

^*^P < 0.05 was considered statistically significant.

^a^Unadjusted baseline values of FPG.

^b^Adjusted for age and sex.

^c^Further adjusted for duration of diabetes, HbA1c, BMI, SBP, AST, eGFR, BUN, SCR,TG, TC, VLDL, FINS, P2INS, HOMA-IR.

^d^Further adjusted for eGFR, BMI, SBP, DBP, BUN, SCR, HbA1c, TG, HDL.

^e^Further adjusted for eGFR, BMI, BUN, SCR, HbA1c, TG.

**Table 7:** Candidate gene association studies for SUA.

**gene**	**genotype**	**SUA (μmol/l)**	*P**-value(allele)**	^***#***^***P*****-value(genotype)**
*ABCG2* (rs2231142)	CC	295.0 ± 117.2	0.0005	0.000
	CA	322.1 ± 122.8		
	AA	323.7 ± 121.5		
*SLC2A9* (rs7660895)	GG	309.6 ± 115.8	0.0438	0.039
	AG	309.3 ± 127.1		
	AA	301.2 ± 116.3		
*SLC2A9* (rs1014290)	TT	311.6 ± 114.0	0.0244	0.040
	CT	307.2 ± 126.8		
	CC	293.1 ± 118.2		

^*^P-value: quantitative association study between serum uric acid levels with alleles of two glucose-uric acid transporter genes, *ABCG2* (rs2231142) and *SLC2A9* (rs7660895 and rs1014290). ^#^*P*-value: comparison of serum uric acid levels among different genotypes.
